# Development and testing of a sports intervention (Girls PLAY)to promote physical activity among rural girls: a feasibility study protocol

**DOI:** 10.1186/s40814-026-01765-2

**Published:** 2026-02-02

**Authors:** Ashleigh Johnson, Jason Bocarro, Emily Kroshus-Havril, Elva Arredondo

**Affiliations:** 1 San Diego State University, 5500 Campanile Drive, San Diego, CA 92182 USA; 2 North Carolina State University, 2820 Faucette Drive, Raleigh, NC 27606 USA; 3 Seattle Children’s Research Institute, 1920 Terry Avenue, Seattle, WA 98101 USA

**Keywords:** Youth sports, Physical activity, Feasibility study, Self-determination theory, Rural, Children, Female

## Abstract

**Background:**

Few United States youth meet physical activity (PA) guidelines, with notable gender, racial/ethnic, and geographic disparities. Sport is one of the best strategies for increasing PA levels, yet girls drop out at a higher rate than boys, and both rural and Hispanic girls participate in lower numbers than their counterparts. Physical literacy (lifelong ability to move) and sport sampling (multiple sport engagement) are core elements of healthy youth sport participation. Commonly cited barriers to starting and/or sustaining sport participation include lack of competence (feeling capable), autonomy (feeling independent), and relatedness (feeling connected to others), in line with self-determination theory (SDT). Unique cultural factors also influence PA and sports participation among Hispanic girls. The proposed research aims to determine the feasibility of an out-of-school sport sampling and physical literacy intervention (Girls PLAY) on rural, Hispanic girls’ PA levels.

**Methods:**

For Aim 1, we will conduct qualitative interviews (*n* = 37) with rural-dwelling girls, parents, coaches, and program staff to identify sport participation determinants for rural, Hispanic girls and use these findings to inform Girls PLAY program development. For Aim 2, we will optimize the program using human-centered design (HCD) strategies such as live prototyping. Staff will implement the program for 2 weeks at a time to a program site, with feedback collected via direct observation and interviews. Feedback will inform additional program modifications. For Aim 3, we will determine the feasibility of the modified program. Staff will deliver the Girls PLAY program using SDT-based instruction at two out-of-school programs. In a sample of thirty girls, we will examine feasibility (recruitment, assessments completed, acceptability, appropriateness, attendance) and pre-post changes in PA, physical literacy, and sport participation, as well as theorized program mediators of SDT constructs.

**Discussion:**

This study’s innovative use of HCD strategies will help culturally tailor the Girls PLAY intervention components and ground this work in knowledge about the rural, predominantly Hispanic border populations and the contexts in which it will be delivered. This work is significant in that addressing barriers to physical activity and sport via an out-of-school program can reduce gender, racial, and geographic disparities in youth activity levels.

**Trial registration:**

ClinicalTrials.gov, NCT06229457, registered January 11, 2024, https://clinicaltrials.gov/study/NCT06229457.

**Supplementary Information:**

The online version contains supplementary material available at 10.1186/s40814-026-01765-2.

## Background

Meeting youth aerobic physical activity (PA) guidelines, defined as at least 6 min/day of moderate- to vigorous-intensity PA (MVPA), is associated with healthy weight status, cardiorespiratory and muscular fitness, and academic performance; and a reduced risk of depression [1–4]. Still, less than 30% of elementary-aged youth meet PA guidelines [5]. There are well-established disparities in PA by gender, with lower PA levels consistently found among girls versus boys [5]. Lower activity levels are also seen among rural versus urban and/or suburban youth [6–9], including within border regions (e.g., United States–Mexico border) [10], and rural youth are 35% less likely than urban youth to meet PA guidelines [11].

Sport is one of the best investments for promoting youth PA [12]. Youth who participate in sport are 64% more likely to meet PA guidelines [13, 14], and are more likely to have better cardiometabolic health in adulthood [15, 16]. However, less than 40% of youth aged 6–12 play sport regularly [17], and gender, geographic, and ethnic disparities persist. Girls drop out at twice the rate of boys by age 14 [18], and both rural and Hispanic girls participate in lower numbers and enter at a later age than their counterparts [5, 19].

**Fig. 1:**
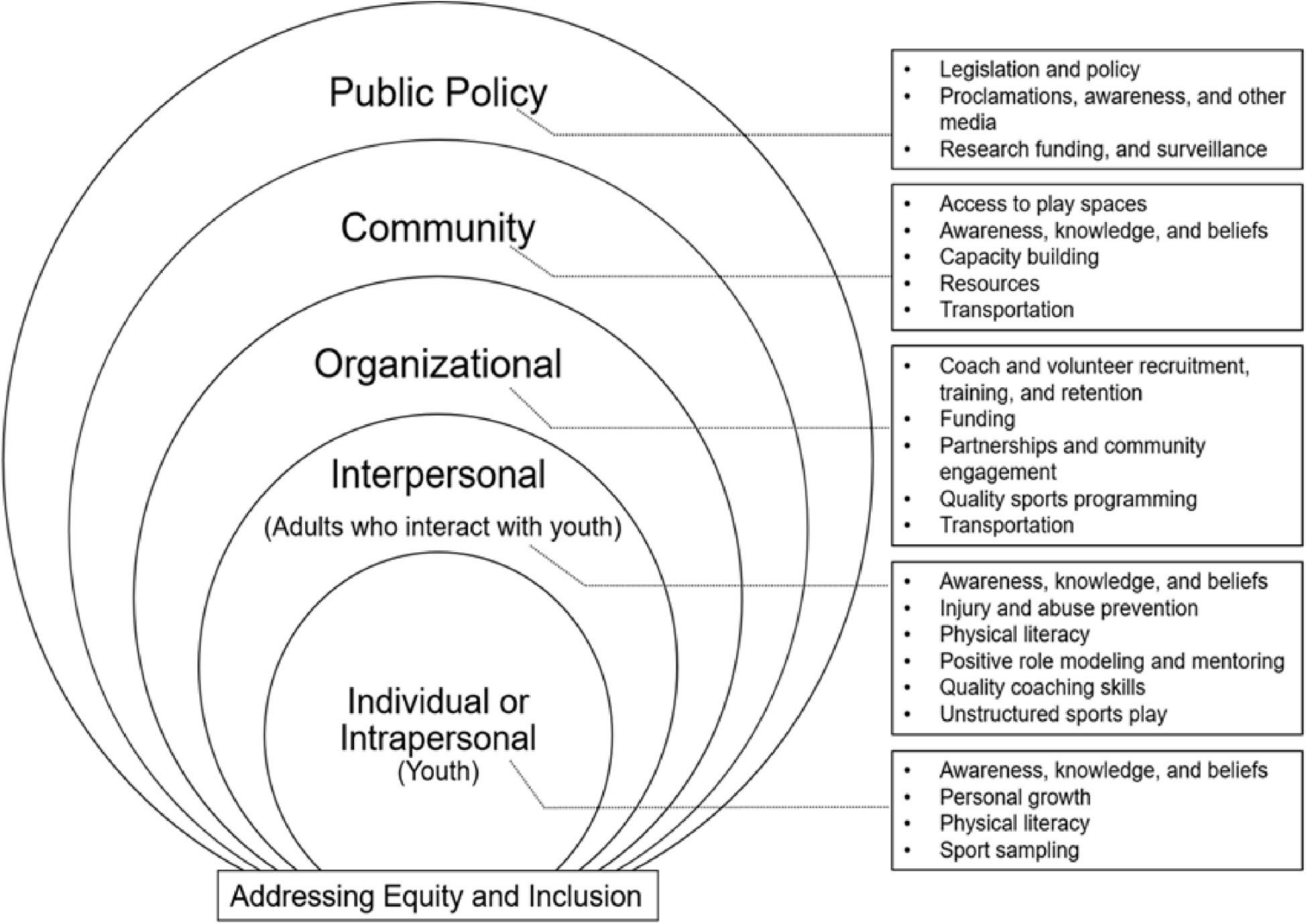
United States Department of Health and Human Services’ Framework for Understanding Youth Sports Participation

The United States Department of Health and Human Services created a framework (Framework for Understanding Youth Sports Participation) based on the social-ecological model to organize determinants of youth sports participation and provide a framework that stakeholders can use to promote youth sports [20, 21]. The framework highlights the need to address multi-level barriers in order to increase youth sports participation (Fig. 1). Each level highlights opportunities to positively influence sport participation while considering how cultural context fits into the framework. Multilevel interventions (i.e., approaches that consider influences at more than one level) are considered to be more effective than single component interventions (e.g., individual-level factors only) [22]. Addressing sport participation factors at the child level as well as the environmental level (intrapersonal, organizational, etc.) will be more likely to impact youth behavior.

Physical literacy is one of the most important elements in healthy, developmentally appropriate youth sport participation and PA [20]. Physical literacy is defined as when an individual has the physical competence (basic movement skills), knowledge, confidence, and motivation to be physically active throughout the life course [23]. Physical literacy and sport skill development can be improved when youth participate in a variety of sports (i.e., sport sampling) [20, 24]. Sport sampling (i.e., engaging in a variety of sports) is strongly recommended for youth under age 12 [25], reduces injury and burnout [26, 27], and provides opportunity to explore different movement types and develop a range of physical skills [20]. Further, physical literacy and sport sampling are guiding principles of the American Development Model, which is used to promote appropriate athlete development and sustained PA throughout the life course [28].

Rural areas face barriers around available sport options, qualified coaches, facilities, and school sport programs [29–33], and thus may experience fewer opportunities for sport sampling. Hispanic girls also report lack of social support for activity and a lack of confidence in their ability [34, 35]. Many individual-level determinants of sport participation align with self-determination theory (SDT), which posits that three fundamental needs (autonomy, competence, and relatedness) are the source of intrinsic motivation [36]. Higher levels of relatedness, autonomy, and perceived competence have been associated with sustained sport participation [37–39]. Further, SDT-centered instruction is associated with higher levels of motivation, self-esteem, and satisfaction of basic needs in a variety of sport settings [40–42].

To date, few interventions have been designed to address multi-level determinants of sports participation among rural, Hispanic youth and their families [43, 44]. Some sport promotion interventions among girls have demonstrated improved PA-related enjoyment and self-efficacy, but these programs were developed outside the USA, focused on adolescent (versus younger) girls, and/or were conducted among predominantly non-Hispanic, urban or suburban youth [45, 46]. In addition, multi-sport programs or camps for youth offered by many community-based organizations (e.g., YMCA) often lack theoretical underpinning, are focused primarily on individual-level determinants of sport participation (e.g., sport sampling, enjoyment); are not designed around Hispanic, rural girls who have unique social and cultural needs; and are mostly focused on older girls (aged 11 +) [47–50]. Further, the efficacy of these programs has not been evaluated. These programs often lack theoretical underpinning, so it is difficult to determine whether the programs are addressing targets likely to lead to behavior change. Thus, their impact on PA and sport participation determinants are largely unknown. There remains a paucity of information on the unique determinants of sport participation and cultural needs among rural, Hispanic girls. Identifying sport participation barriers among this population can help to further inform and culturally tailor interventions designed to promote sustained sport participation.

The purpose of this study is to develop, tailor, and examine the feasibility of a sport sampling and physical literacy out-of-school program among rural-dwelling girls. Study aims include (1) develop a tailored sports sampling, physical literacy intervention through adaptation of existing multi-sport curricula, (2) optimize the intervention using live prototyping, (3) determine the feasibility of the intervention, and (4) characterize the preliminary impact of the program, including pre-post changes in youth PA.

## Methods

### Overview

**Fig. 2:**
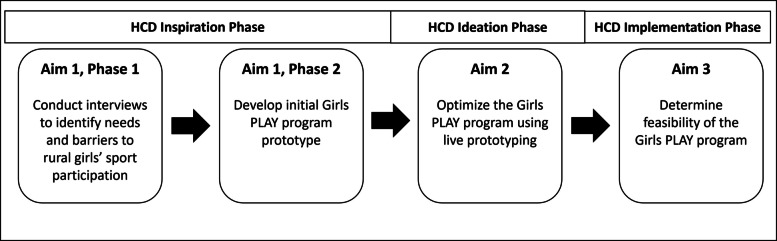
Girls PLAY study aims. *HCD* human-centered design

Figure 2 outlines the study aims. In Aims 1–2, we propose to develop and refine a sport sampling and physical literacy intervention to promote PA among Hispanic girls ages 8–10 in rural areas, entitled “Girls Positive Learning Activities for Youth,” or “Girls PLAY.” We will adapt existing multi-sport curricula to develop the Girls PLAY intervention. We are focusing on girls ages 8–10 years because targeting younger girls leads to more enduring sport involvement [51, 52], and evidence suggests that the prepubescent period represents an age when preferences and motivation for PA and sport are established [53, 54]. In Aim 3, we will examine the feasibility of the Girls PLAY intervention. Secondary outcomes of PA levels, physical literacy, SDT constructs, and sports participation will also be assessed.

### Human-centered design approach

This study will use human-centered design (HCD) strategies to develop and refine the Girls PLAY intervention [55]. HCD supports development of an intervention alongside of—rather than “for”—underserved populations for which they are being designed [56], and is a practical method for operationalizing implementation strategies to support translation of evidence into practice [57]. The Inspiration Phase (phase 1) uses strategies to learn about the intervention context (e.g., target population, their needs, etc.) [55]. During the Ideation Phase (phase 2), the ideas are evaluated and improved [55]. Finally, the Implementation Phase (phase 3) involves measuring relevant outcomes (e.g., feasibility, preliminary effects) [55].

### Setting and population

This study will be based at Boys & Girls Clubs (BGC) sites in Imperial County, California. BGC is one of the largest youth development organizations in the USA, and their goal is to provide safe, positive learning environments for youth that promote good character and healthy lifestyles [58]. Imperial County is a rural region in southeastern California, bordering Mexico to the south, Arizona to the east, and San Diego County to the west [59]. Imperial County has a population of about 180,000, of whom 85% identify as Hispanic or Latino, 75% speak a language other than English at home, and over 17% are considered persons in poverty [10].

## HCD inspiration phase: aim 1—develop a tailored sports sampling, physical literacy intervention

### Aim 1 part 1: identify needs and barriers to rural girls’ sport participation

In Aim 1 Part 1, the research team will conduct individual, semi-structured interviews with girls, parents/guardians, coaches, and BGC staff/leadership who live and/or work in Imperial County, California. We will conduct about 37 individual interviews via Zoom or in-person. The target sample size of 37 interviews was established to achieve thematic saturation across multiple levels of influences [60]. We aim to have 15–20 youth interviews, 15–20 parent interviews, and 3–5 coaches and staff. We will focus on youth and parent interviews because youth will be the end-users and parents serve as the primary gatekeepers for youth activity [61], and 9–17 interviews has been shown to be sufficient for reaching meaningful saturation [60]. Coaches and staff can be considered a more homogeneous group operating under the same institutional protocols, facilities, and logistical constraints, and therefore a smaller sample size is appropriate to avoid informational redundancy [62]. We will develop interview guides for each interview type (parent, child, coach, staff). An overview of interview guide topics are presented in Appendix 1. Objectives of the interviews will be to identify (1) determinants promoting or discouraging sport participation by girls in rural settings, (2) best practices for implementing and sustaining programming in these settings, and (3) how these elements may relate to each other and be incorporated into the Girls PLAY program. Interviews will last 45–60 min and be conducted in English or Spanish as preferred by the participant. Participants will receive a $50 gift card.

### Participants and recruitment

There will be a bilingual (Spanish/English) research assistant to help recruit participants. To be eligible to participate in the individual interviews, all participants must (1) live (youth, parents) and/or work (coaches, BGC personnel) in the rural border region of Imperial County, California. Additionally, youth must (1) identify as female and (2) be aged 8–10 at time of enrollment; parents must have a child that (1) identifies as female and (2) is aged 8–10 at time of enrollment; coaches must have experience providing sport programming to girls ages 8–10; and BGC personnel must have experience in the development and/or delivery of youth programs to girls ages 8–10.

We will recruit participants living within Imperial County. BGC leaders will be recruited from within BGC. Girls, parents, and coaches will be recruited from both within and outside of BGC. We will work with Imperial County BGC leadership and use their established outreach methods for recruiting potential parent and youth participants both within and outside of BGC. We will conduct presentations at BGC staff meetings to recruit staff/leadership, and we will distribute study flyers via BGC listservs and post flyers within the BGC sites to recruit parents, youth, and coaches. Coaches will also be recruited via email from community-based sports leagues that offer programs for girls ages 8–10. We will purposively sample coaches of different genders and race/ethnicity. Materials will be available in both English and Spanish.

### Analysis

Audio will be recorded and transcribed verbatim by a transcriptionist. The study investigator and a trained research assistant will review a random sample of interviews to ensure data integrity. The research assistant will be a member of the Imperial Valley community and can help take cultural factors into account during the analysis. This approach will be informed by Bernal et al. regarding potentially culturally sensitive elements (e.g., language, context, concepts) [63]. We will use NVivo software for coding and thematic content analysis [64–66]. We will inductively develop a hierarchically organized codebook for each group based on research goals, topic areas, and initial data review [65]. We will use the Framework for Understanding Youth Sport Participation to help inform codebook development [20]. Transcripts will be coded independently by two research team members. Coder agreement will be assessed by comparing codings of the 2 coders on 3 transcripts per group (12 total) and discussing the differences until reaching an agreement. If needed, the codebook will be modified to accommodate new codes [65]. During syntheses, coded excerpts will be summarized into themes and subthemes. Findings will be used to inform development of the Girls PLAY program (Aim 1 Part 2).

### Aim 1 part 2: initial Girls PLAY intervention development

We will develop the Girls PLAY intervention using existing multi-sport curricula (described below) while incorporating the theory-based components of sports sampling, physical literacy, and SDT-based instruction. Findings from the individual interviews (Aim 1 Part 1) will be used to tailor Girls PLAY, including identifying necessary cultural adaptations (e.g., language of intervention materials, structure of take-home activities, and additional resources provided to staff and parents) and operational feasibility (e.g., out-of-school session timing, structure of “take-home” activities, potential implementation barriers).

### Girls PLAY intervention overview

**Fig. 3:**
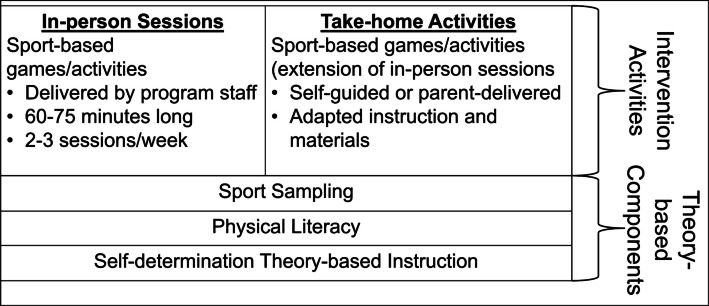
Overview of Girls PLAY intervention components

Figure 3 presents an overview of the Girls PLAY intervention components. The BGC staff will deliver the Girls PLAY program during out-of-school time. The Girls PLAY program will be tailored by findings from the individual interviews, but the core, theory-based components will include (1) sport sampling, (2) physical literacy, and (3) intervention delivery grounded in SDT [36].

Girls PLAY sport sampling and staff-led content, delivery, and length (likely 8–12 weeks) will be designed to build physical literacy and provide opportunities to build autonomy, competence, and relatedness. Findings from the interviews with community members will be used to culturally tailor the intervention while incorporating elements to address multi-level determinants of sport participation (e.g., fun/enjoyment, coaching behaviors).

### Girls PLAY components

The Girls PLAY program will include (1) a series of in-person sessions, led by BGC staff at the BGC sites, and (2) take-home activities.

#### In-person sessions

Based on existing out-of-school programming, we anticipate that in-person sessions will be designed to be 60–75 min and occur 2–3 ×/week, with session frequency/duration to be informed by program staff, parents, and youth. Consistent with the American Development Model’s guidance for sport skill development [28], in-person sessions will center around a different developmentally appropriate sport each week (e.g., soccer, tennis, basketball) [67]. Lesson plans will outline games/activities in which youth engage in fundamental, sport-specific movement skills (e.g., kicking, catching, throwing, etc.) [67], and increase their competence [68]. Lesson plans will be delivered by BGC leaders using acts of instruction derived from SDT (described below).

#### Take home activities

Take home activities will also be developed, consistent with a multilevel intervention approach and to support PA in the home environment. Take home activities will (1) summarize and add on to the week’s in-person sessions and (2) be adapted for delivery by parents and/or self-guided participation by youth. Take-home activities will provide information for engaging in the past week’s activities (materials needed, instructions, autonomy supportive instruction for parents) as well as additional activities that parents and youth can participate in together. Additional content will be considered based on parents and youth feedback (e.g., culturally relevant activities). To address the home environment, the “take-home” component of Girls PLAY follows evidence-based strategies for family-based PA interventions. Research demonstrates that multi-component interventions, including those that address parental physical activity practices and home-related determinants, are more likely to be effective [69, 70]. By including take-home activities and supporting materials, we aim to bridge the gap between out-of-school sessions and the home environment. Recognizing the potential challenges in implementation, the feasibility of this component will be assessed through interviews to inform refinements of Girls PLAY. All materials will be available in English and Spanish and will be written between a 3rd to 5th grade reading level. Delivery mode for take-home activities (e.g., email, mailings) will be based in existing BGC channels of communication and further informed by community member feedback collected during Aim 2.

### Development of the initial Girls PLAY prototype

The initial Girls PLAY program prototype will be developed through adaptation of existing multi-sport curricula using the following steps: (1) publicly available sport sampling curricula will be examined (e.g., YMCA Physical Education Program, Jr. NBA Physical Education) [50, 71]; (2) curricula session activities will be narrowed down based on their focus on youth ages 8–10 and inclusion of developmentally appropriate fundamental movement skills [67]; (3) the remaining activities will be grouped by sport, with the goal of at least 25 activities/sport (e.g., dribbling red light/green light; throwing at targets) and at least 5 different sports, and evaluated to ensure that a range of fundamental movement skills are represented [67]; and (4) activities will be culturally adapted as needed (e.g., language, content, and context) [63], with a focus on incorporating feedback from interviews with members of the community.

### Girls PLAY delivery training

Research team members will train BGC leaders at the selected BGC sites in intervention delivery that will include didactic instruction in program objectives and protocol, as well as intervention materials. BGC leaders will need to demonstrate an understanding of program principles and an ability to deliver in-person sessions prior to delivery, including experience and/or potential for program delivery among Hispanic girls ages 8–10. Table 1 provides the core leader competencies that the intervention delivery training will be built on, with examples of instructional behaviors that are rooted in SDT [72, 73]. Feedback collected from community members during live prototyping (Aim 2) will ensure that the program content is delivered in a culturally sensitive manner.

**Table 1:** Core competencies for Girls PLAY delivery training by Boys and Girls Clubs staff

Training core competency	Examples
Nurture inner motivational resources	Encourage youth to set meaningful goals for themselves
Rely on noncontrolling language	Provide information about different options within the activity
Provide explanatory rationales	Explain how the activity can help youth meet their goals
Display patience to allow time for self-paced learning	Provide time for youth to work through activity in their own way
Acknowledge and accept negative affect during activities	Encourage youth to voice their opinions

### HCD ideation phase: aim 2—optimize the Girls PLAY program using live prototyping

For Aim 2, we will conduct live prototyping of the initial Girls PLAY prototype among BGC sites. This testing is intended to identify ways to improve feasibility, usability, acceptability, and impact of Girls PLAY [74]. Live prototyping involves the process of making new ideas sufficiently tangible in a real-world setting to quickly test their value [75], and places end-users at the center of development. The key deliverable will be a final Girls PLAY prototype.

### Recruitment strategy and approach

We will recruit a rural-serving BGC site within Imperial County to conduct live prototyping and refinement of the initial Girls PLAY prototype. We will work with BGC leadership to select their preferred site. We will provide the selected BGC site with 1–2 week blocks of curriculum materials (i.e., one sport module), rather than the entire program at once, to support prototyping and refinement of the intervention. These materials will be grounded in SDT and include protocols and materials for the in-person sessions and take-home activities. BGC intervention delivery training will also be provided by a research team member to conduct the intervention portion provided. This process will be revisited with additional curriculum blocks until the entire program has been implemented and refined based on community member feedback.

### Participants

To be eligible to participate in the live prototyping, youth participants must (1) identify as female, (2) be aged 8–10 years old at time of enrollment, and (3) enrolled at a BGC site conducting live prototyping of the intervention (Girls PLAY). Parents must have a child that (1) identifies as female, (2) is aged 8–10 at time of their enrollment, and (3) enrolled in a BGC site conducting live prototyping of the intervention; and BGC personnel must be located at a BGC site conducting live prototyping of the intervention.

### Data collection

We will collect data on program staff, parent, and youth perspectives via (1) direct observation and (2) semi-structured interviews. Direct observation of two in-person sessions and one BGC training per cycle (3–4 anticipated) will be made by research team members using the adapted Cheffers’ Adaptation to Flander’s Interaction Analysis System [76, 77] to examine whether learning objectives were met and note deviations from the protocol [78].

Individual interviews will be conducted with BGC personnel after in-person session observations, as well as opportunity samples of parents and youth after at least one week of the Girls PLAY program has been delivered. Interview topics will be finalized upon completion of Aim 1 interviews to reflect specific usability/feasibility concerns, but we anticipate the following topic areas for interview questions: elements that were liked or not liked; enhancements to consider for further adaptation, including cultural adaptation [63]; knowledge and beliefs about Girls PLAY; perceived benefit from the intervention; and implementation of Girls PLAY program components. Interviews will last 15–25 min and be conducted in English or Spanish as preferred by the participant. Participants will receive a $45 gift card.

## HCD implementation phase: aim 3—determine feasibility of the Girls PLAY intervention

### Overview

Once the initial Girls PLAY prototype has been developed (Aim 1) and refined through live prototyping (Aim 2), we will conduct a single-arm feasibility study with pre-post intervention measures to assess the research and intervention process, including preliminary examination of participant responses to the intervention [79, 80].

### Setting and participants

We will randomly select two BGC sites in Imperial County, California to receive the final Girls PLAY program prototype, with the goal of enrolling 30 youth participants across both sites. To be eligible, the BGC site must be (1) located within Imperial Valley and (2) provide programming to girls ages 8–10. To be eligible to participate in the feasibility study, youth participants must (1) live in Imperial County, California; (2) identify as female; (3) be aged 8–10 years old at time of enrollment; and (4) enrolled at a BGC site participating in the feasibility study.

Data collection will occur at baseline (0 weeks) and post-intervention (week 10–12). All measures will be available in English and Spanish. Recruitment strategies will include BGC’s established outreach methods for recruiting potential parent and youth participants, including sharing study flyers via BGC listservs and within the BGC sites. The research team will also present during BGC staff-parent meetings to share more information about the study.

### Data collection

Data collection will include electronic or in-person surveys for youth, parents, and staff (pending preferences elicited in formative work); direct observation; device-based PA assessment; and use of administrative data. Table 2 provides an overview of study variables, assessment methods, and collection time point. We will use REDCap to distribute the survey links to participants. Surveys will be available in English and Spanish.

**Table 2:** Single-arm feasibility study data collection

Variables	Assessment method	Time
**Primary outcomes**		
Recruitment capability	Administrative data	F/U
Data collection procedures	Administrative data	F/U
Intervention acceptability	Parent and Staff Surveys	F/U
Intervention appropriateness	Parent and Staff Surveys	F/U
Intervention feasibility	Parent and Staff Surveys	F/U
Participant attendance	Administrative data	F/U
**Secondary outcomes**		
Physical activity	Youth-worn Accelerometer	B, F/U
Physical literacy	Youth survey, Direct observation	B, F/U
SDT constructs	Youth survey	B, F/U
Sport participation	Youth survey	B, F/U
**Additional variables**		
Demographics	Staff, parent, and youth surveys	B

*F/U* follow-up, *B* baseline, *SDT* self-determination theory

BGC staff will receive $50 for completing post-intervention surveys and collecting administrative data throughout the intervention. Parents will receive $15 for completing pre- and post-intervention surveys. Youth will receive $50 for completing surveys pre- and post-intervention and an additional $50 for wearing the accelerometers for 1 week.

### Study outcomes

#### Primary outcomes

The primary outcomes, based on the essential components of a feasibility study [81], will be (1) recruitment capability, (2) data collection procedures and outcome measures, (3) acceptability and suitability of the intervention and study procedures, and (4) preliminary participant response to Girls PLAY, including changes in key outcomes.

*Recruitment capability* will be measured via administrative data, with a feasibility metric of successfully enrolling 30 participants in the Girls PLAY program. *Data collection procedures* will be assessed, in part, by the number of study assessments completed, with a feasibility metric of at least 75% of participants completing assessments at both baseline and follow-up [82, 83]. *Acceptability* of the intervention will be measured via the Acceptability of Implementation Measure (AIM) [84]. *Appropriateness* of the intervention will be measured via the Intervention Appropriateness Measure (IAM) [84]. *Feasibility* of the intervention will be measured via Feasibility of Intervention Measure (FIM) [84]. The AIM, IAM, and FIM are written at a 5th grade level and have demonstrated strong psychometric properties [84]. The feasibility metric for the AIM, IAM, and FIM will be a mean score of ≥ 3. *Participant attendance*, a measure of preliminary evaluation of participant response, will be measured using administrative data, with a goal of participants attending, on average, ≥ 75% of in-person sessions [82, 83].

#### Secondary outcomes

To assess feasibility of collecting outcome data and preliminary changes in outcome measures (outcome 4), we will collect data on (1) PA levels, (2) physical literacy, (3) SDT constructs, and (4) sport participation at baseline (week 0) and post-intervention (week 10–12).

#### Physical activity

We will use Actigraph GT9X-BT (ActiGraph LLC, Pensacola, FL) to obtain a valid, objective measure of youth PA. We will examine minutes spent in moderate- to vigorous-intensity PA (MVPA). Accelerometers will be on an elastic belt and placed above the iliac crest of the hip. Data will be collected over 7 days (sample frequency of 30 Hz; 15-s epochs) during waking hours. We will use appropriate cut points to classify the time youth spent in respective activity intensities [85]. A research staff member will be onsite at the BGC to assist with placing the device on youth on day one and collecting the device on day seven.

*Physical literacy* changes among youth will be assessed using accelerometers and the Canadian Assessment for Physical Literacy, Second Edition (CAPL-2) [86, 87]. CAPL-2 measures physical, affective, and cognitive domains of physical literacy among ages 8–12 via three Physical Competence protocols (plank, Progressive Aerobic Cardiovascular Endurance Run [88], Canadian Agility & Movement Skill Assessment [89]), two Daily Behavior protocols (pedometer, self-reported PA), and a 22-item questionnaire assessing knowledge, motivation, and confidence (validated among ages 8–12) [86]. CAPL-2 allows for delivery of select individual protocols [86], and we will use accelerometers in place of pedometers and self-reported PA.

### SDT constructs

To measure changes in SDT constructs, we will conduct youth surveys. We will use the 9-item Autonomy-Supportive Coaching Questionnaire (ASCQ) to measure autonomy [90]. The ASCQ has been validated in ages 7–18, with α = 0.88 and α = 0.84 for internal consistency of interest in athlete’s input and for praising autonomous behavior, respectively [91]. Although the Spanish version of the ASCQ has not been validated among the age group of interest, it has been validated among Spanish athletes ages 12–17 and was found to have adequate temporal stability and internal consistency [92]. We will use an adapted version of the Perceived Competence Scale (4-item, PCS) to measure competence [36]. The PCS has a high face validity among children in a sport and PA context [93, 94]. Reliability of the perceived competence items was α = 0.90 [95]. We will use the 5-item acceptance subscale of the Need for Relatedness Scale (NRS) to measure relatedness [96]. The NRS has demonstrated adequate construct validity and internal reliability in research among young athletes [97, 98].

### Sport participation

Sport engagement will be assessed via youth survey examining current sport involvement (i.e., length of involvement, primary and secondary sport) and behavioral intention (e.g., “I intend to play sports this year”) adapted from items used in studies among similar age groups [99, 100]. Sport enjoyment will be measured using the adapted 5-item Enjoyment subscale of the Sport Commitment Questionnaire-2 [101–103]. Internal consistency and confirmatory factor analyses support reliability and validity among youth [103–105].

### Additional variables

In addition to the primary and secondary variables, we will also collect youth, parent, and staff demographic information via survey. We will collect youth demographic information such as age, race/ethnicity, and prior sports participation. We will also collect parent (e.g., sex, age, race/ethnicity, highest level of education, and household income) and staff (e.g., sex, age, race/ethnicity, number of years as a BGC staff member, role) demographic information.

### Sample size justification

Feasibility studies are not designed or powered to detect statistically significant effects on outcomes, but rather to assess whether key study processes are feasible. The target sample size of 30 participants for the feasibility study (Aim 3) was determined based on sample size recommendations for feasibility studies that typically range from 20 to 50 participants [106]. This sample size will allow us to assess recruitment capability and determine if our target of enrolling 30 participants is achievable; evaluate data collection procedures; examine acceptability, appropriateness, and feasibility measures; estimate variability in outcome measures; and assess participant attendance.

### Data analysis

Given the feasibility nature and sample size of the study, we will report the amount of missing data for each variable, rather than conducting any inferential missing data analysis. For the primary aim of feasibility, descriptive statistics will be used to examine and compare feasibility outcomes with pre-determined benchmarks. For the secondary aim of changes in key outcomes, we will report descriptive statistics (means, standard deviations, medians, interquartile ranges) and 95% confidence intervals for in mean daily minutes of MVPA, physical literacy, SDT constructs, sport enjoyment, and number of sports played at baseline and follow-up. We will also report the proportion of participants engaging in sport at each timepoint with 95% confidence intervals. Accelerometer data will be adjusted for wear time. These estimates of variability and precision will inform the design and sample size calculations for a future RCT. All analyses will be conducted using Stata 17.0 (StataCorp LP, College Station, TX). Analyses will follow intention-to-treat. Based on the quantitative findings, we will consider a sequential explanatory design in which qualitative interviews with a subset of participants are used to help interpret the quantitative results [107].

## Discussion

This study contributes to the field of youth PA promotion in multiple ways. First, we will develop and examine the first evidence-based, multi-level program (Girls PLAY) aimed to promote PA through sport among rural-dwelling, Hispanic girls. The proposed intervention targets Hispanic girls in a rural, border region of California, a historically underserved youth population. We will develop and culturally tailor Girls PLAY alongside this population using HCD strategies. The HCD approach was initially intended for product development and management system design [74], and has only more recently been applied to intervention design, adaption, and implementation [108]. HCD has demonstrated potential to improve behavioral intervention response by more closely aligning evidence-based intervention implementation with the priorities and context of the target population [109]. HCD strategies will be used throughout intervention development to culturally tailor intervention components and ground this work in knowledge about the rural, predominantly Hispanic border populations and the contexts in which it will be delivered [110], thus producing a more usable intervention [111]. By involving community members throughout the intervention design process, we aim to incorporate innovative solutions to the most cited multi-level barriers while culturally tailoring the Girls PLAY program.

Additionally, our partnership with BGC supports sustainability and scalability of the Girls PLAY program and removes barriers around developing new systems and infrastructure. Building new infrastructure around youth sport is costly and time-consuming [112], particularly in rural settings where there are typically fewer available resources [112, 113]. Our partnership with BGC and the proposed program to be developed (Girls PLAY) can easily be expanded and implemented in BGC clubs nationally, and training existing BGC leaders within the community increases sustainability. Beyond the immediate evaluation of the Girls PLAY program feasibility, there is significant potential for this approach to be adapted and scaled across diverse contexts. While this study focuses on rural Hispanic girls, the underlying HCD framework provides a replicable process for tailoring interventions to other populations. Future research can explore the adaptation of the Girls PLAY program for other environments (e.g., urban) and populations (e.g., older age groups, cultural contexts). We plan to present the results of the feasibility study once the program is implemented so we can provide a foundation for future cross-cultural and cross-geographic applications and utilize the established infrastructure of the Boys and Girls Clubs of America to facilitate national dissemination.

There are several potential challenges with this work. To address recruitment and retention issues, we will work with BGC leaders to develop outreach strategies for youth, parents, and BGC staff. This will be impacted by potential staff turnover and staff investment in the program. Another potential challenge is that it may not be feasible to address all barriers identified in the Aim 1 community member interviews. However, findings from these interviews will be disseminated with the research community, which will be an important step in addressing barriers to rural girls’ sports participation and PA. Although interview results may not be generalizable to all rural populations, we will strive to include participants from a variety of rural areas. Further, given the qualitative nature of such work, the goal is not generalizability but achieving a depth of knowledge not easily obtained through quantitative methods.

A limitation of this study is the absence of pre-specified progression criteria to systematically determine whether to proceed to a full-scale randomized controlled trial. While we have established feasibility benchmarks for our primary outcomes (e.g., recruitment, data collection completion, acceptability, appropriateness, feasibility, and attendance), we have not specified a priori thresholds using a traffic light system as recommended by Avery et al. [116] and Mellor et al. [115]. The absence of such criteria limits our ability to make transparent, systematic decisions about progression to a definitive trial and may introduce subjectivity into the decision-making process. Future feasibility studies should incorporate clear progression criteria during the design phase to enhance methodological rigor and transparent reporting of progression decisions.

## Summary

Fewer than 30% of elementary-aged youth meet PA guidelines, with lower activity levels found among girls, racial minorities, and those living in rural areas. Sport is one of the best strategies for promoting PA, yet girls, Hispanics, and rural populations participate in youth sport at lower numbers and drop out at a higher rate. Developing interventions around physical literacy and sport sampling, with intervention delivery grounded in SDT principles, is a promising strategy for promoting youth sport. However, the few existing interventions aimed to promote girls’ PA through sport were developed outside the USA, focused on adolescent (versus younger) girls, and/or conducted among urban or suburban youth, limiting generalizability. The goal of this study is to use HCD strategies to develop a tailored sport sampling and physical literacy intervention, in collaboration with BGC, and examine the impact on girls’ PA. This work is significant in that addressing barriers to PA and sport via BGC can reduce gender, racial, and geographic disparities in youth activity levels.

## Supplementary Information

Supplementary Material 1.

## Data Availability

Not applicable.

## Appendix

### Interview guide question topics

**Table:**

Interview Guide Question Topics (wording adapted for simplicity in this table)	Youth	Parent	Coach	Staff
**Current physical activity and sports participation**				
What are some things that the child does that are physical activity? ● Who are they active with? ● Where are they active?	✔	✔		
Does the child currently play any sports? ● Who do they play with? ● Where do they play?	✔	✔		
What sports organizations is the child involved with?		✔		
Who decides if the child plays sports?	✔			
**Previous sports participation**				
What are some sports that the child has done before that they are not doing now? ● Why did the child stop playing these sports? ● Does the child want to keep playing these sports?	✔	✔		
**Desired sport experience**				
What improvements could be made to girls’ sport opportunities?	✔	✔	✔	
Are there any new sports that the child wants to try?	✔			
What do coaches want girls and families to get out of youth sports?			✔	
**Barriers/Facilitators to sports participation and program delivery**				
What does the child like about playing sports?	✔			
What are some things that make it difficult for the child to play sports?		✔		
What does the child dislike about playing sports?	✔			
What are some things that make it easier for the child to play sports?	✔	✔		
Are there time constraints involved with the child’s sport participation?		✔		
Who in the family plays sports? ● Does the child play sports with them?	✔			
Do any of the child’s friends play sports? ● Does the child play sports with them?	✔			
Are the child’s friends and family who play sports mostly male or female?	✔			
Does the child have any role models they look up to within sports?	✔			
How often does the child watch sports?	✔			
Has the child ever heard the phrase, “Throwing or running like a girl?”	✔			
What types of sports programs are available to the child nearby?		✔		
Do any other children in the family play sports? ● Where does this fit into the parent’s priorities?		✔		
What are the benefits of girls playing sports?		✔		
How concerned are parents with safety in youth sports?		✔		
How do coaches keep players engaged during practice and games?			✔	
How do coaches keep girls involved in sports long-term?			✔	
What challenges do coaches experience when coaching youth sports? ● How do they handle these challenges?			✔	
What are things that make the coaching experience easier?			✔	
What resources have helped with coaching? ● What additional resources would be helpful?			✔	
What are the biggest challenges that organizations face in providing youth/sport programming?			✔	✔
What has been helpful to organizations providing youth/sport programming?			✔	
What financial challenges have sports leagues/organizations experience when trying to provide programming?			✔	
What is the structure of a typical youth program?				✔
What helps a youth program be successful? ● What training or materials are available? ● What additional resources would be helpful? ● What would support program sustainability?				✔
How are families informed about youth programs? ● What are the biggest challenges in communicating with families?				✔
Which youth programs are girls most excited about?				✔
What are effective strategies for keeping girls participating in youth programs?				✔
**Parent and community priorities**				
There are many ways that a parent can care for a child and support them in growing up healthy and safe. Where does PA fit into the list of things that you think about as a parent?		✔		
Do parents want their child to play sports?	✔			
Do parents encourage their child to play sports?	✔	✔		
Are parents able to watch their child play sports?		✔		
Do parents coach or have other roles on their child’s team?		✔		
Outside of sports, what are the most important issues within the community?		✔	✔	✔
How do parents typically get involved with youth programs?				✔
**Coaching**				
What roles have coaches had within youth sports?			✔	
How did coaches get involved with coaching youth sports?			✔	
How would coaches describe their coaching style? ● How do coaches adjust their coaching for different ages and gender?			✔	

## Abbreviations

PA: Physical activity
SDT: Self-determination theory
HCD: Human-centered design
MVPA: Moderate- to vigorous-intensity physical activity
Girls PLAY: Positive Learning Activity for Youth
BGC: Boys and Girls Clubs
AIM: Acceptability of Implementation Measure
IAM: Intervention Appropriateness Measure
FIM: Feasibility of Intervention Measure
CAPL-2: Canadian Assessment for Physical Literacy, Second Edition
ASCQ: Autonomy-Supportive Coaching Questionnaire
PCS: Perceived Competence Scale
NRS: Need for Relatedness Scale
